# Strategic alliances and green innovation bubbles: drivers, distortions, and environmental consequences

**DOI:** 10.3389/fpubh.2025.1688327

**Published:** 2025-10-07

**Authors:** Hongda Wang, Jingyi Xiang, Tianwei Xie

**Affiliations:** Graduated School of Business Administration, Dongshin University, Naju-si, Republic of Korea

**Keywords:** strategic alliances, green innovation bubbles, new-quality productivity, information asymmetry, policy resource acquisition

## Abstract

**Introduction:**

Amid global ecological crises and China’s “dual-carbon” goals, green innovation has emerged as a crucial strategy for sustainable development. However, the phenomenon of “green innovation bubbles”—where innovation input and actual environmental output are misaligned—raises concerns about the effectiveness of such efforts. This study explores how corporate strategic alliances influence the formation of green innovation bubbles and examines the moderating role of new-quality productivity.

**Methods:**

Drawing on resource dependence theory and signaling theory, we constructed a panel dataset of 2,346 Chinese A-share listed companies from 2015 to 2022. We developed a Green Patent Bubble Index (GPBI) based on green patent growth, citation frequency, and structural quality. Regression analyses were conducted to assess the direct, moderating, and mediating effects of strategic alliances, new-quality productivity, information asymmetry, and policy resource acquisition.

**Results:**

Findings indicate that strategic alliances significantly contribute to green innovation bubbles. Horizontal and diversification alliances amplify this effect, whereas vertical alliances mitigate it. New-quality productivity negatively moderates the alliance-bubble relationship, meaning firms with stronger innovation capacities are less prone to bubbles. Information asymmetry and policy resource acquisition both serve as significant mediators in this process. The bubble effect is more pronounced in state-owned enterprises and in regions with weaker environmental regulations.

**Discussion:**

These findings reveal the dual nature of strategic alliances—serving both substantive and symbolic purposes—and highlight the risks of misallocated innovation under policy-driven incentives. The results underscore the importance of firm-level absorptive capacity and regulatory strength in curbing superficial green innovation. This research provides valuable insights for policymakers, businesses, and investors seeking to promote authentic and effective green innovation aligned with environmental and public health objectives.

## 1 Introduction

The global climate crisis is intensifying, and governments around the world are implementing strict environmental policies to drive green transformation in businesses. China’s “dual carbon” targets have further elevated green innovation to a core position in corporate strategy (1). Despite rapid growth in investments in green innovation, the improvement in environmental quality has been disappointing, and breakthroughs in green technologies are far below expectations (2). This paradox suggests the existence of a “bubble” in green innovation—where the input (investment) and output (environmental benefits) are severely mismatched (3). The green innovation bubble not only leads to a serious misallocation of scarce resources but may also trigger a series of social issues: diminishing returns on green innovation policies, a decline in market trust in environmentally friendly businesses, and even hindering truly valuable green technology breakthroughs, ultimately jeopardizing the achievement of climate goals (4).

Given the critical role that green innovation plays in achieving global sustainability goals, understanding the formation mechanism of the green innovation bubble is crucial. Although green innovation is widely regarded as a key pathway to reducing environmental degradation and supporting ecological resilience (5), recent empirical findings reveal a disconnect between rising levels of green innovation investment and corresponding improvements in environmental performance, suggesting the presence of a “bubble” in green innovation—characterized by a significant mismatch between innovation input and output (2, 3). This phenomenon not only reflects inefficiencies in resource allocation but may also weaken public and investor confidence in green initiatives, thereby jeopardizing the achievement of climate goals and long-term public health benefits (4–7).

A growing body of research has examined the drivers of green innovation, such as government regulation, fiscal incentives, and firms’ organizational learning capacity (1, 8). Among these, strategic alliances have emerged as a critical organizational form enabling firms to access complementary resources, reduce R&D uncertainty, and accelerate the development of green technologies (9–12). Alliances are believed to enhance firms’ environmental innovation capability by promoting knowledge flows and resource co-investment across organizational boundaries.

Recent research also highlights that not all alliances yield substantive innovation outcomes. Under mounting institutional pressure, some firms may form alliances primarily to signal green commitment rather than to achieve genuine technological breakthroughs, thus contributing to symbolic or superficial environmental innovation (13–15). This dual function of strategic alliances—both as innovation facilitators and as symbolic tools—raises an important question: why do some alliances lead to real green value, while others contribute to green innovation bubbles? Existing research has shown that under pressure from environmental policies and market expectations, firms may leverage alliances to enhance their environmental legitimacy rather than to drive genuine innovation (16).

Yet, few studies have systematically investigated how strategic alliances contribute to the formation of green innovation bubbles, and under what conditions such symbolic behavior arises (17, 18). Most existing literature focuses on the positive effects of alliances on innovation, while theoretical mechanisms underlying their distortive effects—particularly the interplay between resource dependence and signaling dynamics—remain underexplored (19–21). Moreover, the internal heterogeneity among firms—such as innovation absorption capacity—has not been adequately considered when assessing alliance outcomes. In particular, the potential moderating role of new productive forces in converting alliance-based collaboration into substantive innovation has been largely overlooked in prior studies (22–24).

This study proposes an analytical framework that integrates the concept of new productive forces as a moderating variable alongside resource dependence theory (25) and signaling theory (26). New productive forces are defined as modern technological capabilities and high-end industries focused on green low-carbon features, which are deeply integrated with the real economy (23). These forces emphasize the transformation of innovation into tangible outcomes. Firms with stronger new productive forces are better able to leverage the resources from strategic alliances to generate meaningful green innovation, thereby reducing the risk of green innovation bubbles. This framework provides a multi-dimensional approach to understanding how strategic alliances and new productive forces interact to shape green innovation outcomes.

In addressing these issues, this research seeks to answer three key questions:

- How do corporate strategic alliances contribute to the creation of green innovation bubbles?
- How do new productive forces moderate this relationship?
- What roles do information asymmetry and policy resource acquisition play in this process?

The study uses empirical data from China’s A-share listed companies between 2017 and 2022 to answer these questions.

This study makes several important theoretical contributions. First, it uncovers the “dark side” of strategic alliances, showing that under certain conditions, alliances may inadvertently create green innovation bubbles. This challenges the traditional view that strategic alliances always promote genuine innovation and offers a fresh perspective on how corporate collaboration can sometimes hinder real environmental progress. Second, the study introduces new productive forces as a critical moderating variable, bridging a gap in the literature by explaining why similar strategic alliances can yield different outcomes depending on the company’s capacity for meaningful innovation. Third, the study reveals the micro-mechanisms behind green innovation bubbles, specifically the roles of information asymmetry and policy resource acquisition in fostering these bubbles. This deeper understanding of how and why green innovation bubbles form can help inform both policy and business strategies aimed at promoting long-term sustainability and public health. Finally, it distinguishes the impacts of different types of strategic alliances horizontal, vertical, and diversification alliances offering a more refined approach to understanding their effects on green innovation.

The practical implications of this study are also relevant. For businesses, the findings offer insights into how to identify and manage the risks associated with green innovation bubbles in strategic alliances, helping to optimize alliance selection and management strategies. For policymakers, the research suggests ways to refine green innovation policies, ensuring that incentives are aligned with substantial environmental and public health outcomes, rather than superficial or temporary gains. For investors, the study provides guidance on how to assess the true value of corporate green innovation, helping to avoid capital misallocation and supporting investments that contribute to long-term sustainability.

## 2 Theoretical foundation and research hypotheses

While strategic alliances are widely recognized for their role in enhancing green innovation by facilitating resource sharing and inter-organizational learning (10–12), recent studies have raised concerns about their potential unintended consequences. In particular, the growing divergence between green innovation input and actual environmental or technological output—referred to as the “green innovation bubble”—has drawn attention to the symbolic functions these alliances may serve under institutional pressure (2, 14).

Prior literature emphasizes both the advantages of alliances in addressing environmental uncertainty (8, 15) and the risks of superficial engagement in sustainability initiatives (13). However, limited attention has been paid to how the structure and signaling mechanisms of strategic alliances may contribute to the formation of green innovation bubbles, particularly under conditions of information asymmetry and policy-driven incentives (18). While it is acknowledged that alliances can sometimes fail to deliver meaningful innovation, a systematic theoretical framework explaining the dual role of alliances—both substantive and symbolic—remains underdeveloped (6).

Addressing this gap, the present study proposes a theoretical framework that integrates Resource Dependence Theory and Signaling Theory to explore the conditions under which strategic alliances may contribute to the inflation of green innovation bubbles. Specifically, we argue that firms, driven by a need to secure external resources and convey environmental commitment to stakeholders, may—under information asymmetry—be incentivized to prioritize symbolic over substantive innovation. This perspective shifts the discourse from assessing the general efficacy of alliances to examining the contingencies under which they may produce counterproductive outcomes in the context of green innovation.

### 2.1 A dual-theory framework: resource dependence and signaling

To dissect the complex relationship between strategic alliances and green innovation bubbles, this study adopts a dual theoretical lens, integrating Resource Dependence Theory (RDT) and Signaling Theory (ST). We argue that neither theory alone is sufficient, but together they provide a powerful explanatory framework. RDT is the optimal choice for explaining the motivation behind forming green alliances. It posits that organizations are dependent on their external environment for critical resources and will engage in strategies, like alliances, to manage this dependency and reduce uncertainty (27). In the context of green innovation, these resources include specialized technology, capital, and crucial policy support (28). Thus, RDT explains why firms seek partners.

However, RDT primarily focuses on the substantive acquisition of resources and does not fully account for the symbolic and informational aspects of strategic actions in a market with imperfect information. This is where Signaling Theory becomes essential. Signaling Theory asserts that in the presence of information asymmetry, firms use observable actions to convey private information about their quality and prospects to external stakeholders (26). Forming a green strategic alliance is a highly visible and potent signal. It communicates a firm’s commitment to sustainability and its innovative capabilities to investors, customers, and regulators (29), potentially unlocking market recognition and policy favor.

By integrating these two theories, we build a more comprehensive logic. RDT explains the firm’s internal drive for resources, while ST explains the external perception and market reaction to the alliance. The tension between these two forces is what can give rise to a green innovation bubble. Firms may be motivated by resource needs (RDT) but find it easier or more immediately rewarding to focus on the signal (ST), especially when the true quality of green innovation is difficult to assess. This dual framework allows us to hypothesize not only that alliances can lead to bubbles but also to explore the mechanisms (information asymmetry, resource acquisition) and boundary conditions (firm capabilities, alliance type) that shape this relationship.

### 2.2 Corporate strategic alliances and the green innovation bubble

Strategic alliances are cooperative relationships established to achieve shared goals. In the context of environmental sustainability, however, we argue that they can, on average, contribute to a “green innovation bubble”—a severe mismatch between a firm’s green innovation inputs and its substantive outputs (30). This phenomenon has been empirically observed as a “decoupling” between R&D expenditures and meaningful patents in collaborative ventures (31, 32). Grounded in resource dependence and signaling theories, we first propose an overall effect.

From a resource dependence perspective, the pursuit of resources can lead to unintended negative consequences (33). Firms facing strong environmental policy pressure may form alliances to access subsidies or attract capital without a genuine intent to innovate, leading to a bubble where resources are secured but not effectively utilized (34). This can also foster “collusive inefficiency” or “free-riding,” further widening the input–output gap (35, 36). From a signaling perspective, alliance announcements serve as powerful, positive signals that can inflate market perceptions of a firm’s green capabilities, often confirmed by short-term positive stock reactions (37). To maintain this perception, firms may feel compelled to announce more projects, perpetuating the bubble regardless of substantive progress (38).

Considering these prevalent pressures for symbolic action and inefficient resource acquisition across many alliance forms, we posit an overall positive relationship.

*Hypothesis 1:* Corporate strategic alliances, in aggregate, are positively correlated with green innovation bubbles.

However, this overall positive relationship is not uniform and masks significant differences based on the type of alliance. Corporate strategic alliances are not monolithic; following Lavie (39), they can be disaggregated into horizontal (intra-industry), vertical (supply chain), and diversification (cross-industry) types, each with distinct motivations that alter their impact on the green innovation bubble (40).

Horizontal alliances, formed between competitors, are fraught with competitive tension (41). This dynamic can incentivize firms to engage in symbolic innovation races, leading to excessive investment simply to signal industry leadership, thereby exacerbating the bubble risk (27).

Diversification alliances, while offering access to heterogeneous knowledge, often suffer from high coordination costs and severe information asymmetry (42). The novelty of cross-industry collaboration carries a strong signaling effect, potentially tempting firms to prioritize the symbolic value of the alliance over achieving substantive outcomes, thus increasing the likelihood of a bubble (43).

In stark contrast, vertical alliances integrate firms across the supply chain. This structure inherently grounds innovation in practical application and market needs (15). Empirical studies show that vertical collaborations in sectors like renewable energy often lead to more commercially viable products and process improvements due to direct market feedback loops (44). This focus on tangible efficiencies and concrete outcomes promotes substantive innovation and reduces the tendency for superficial R&D (12, 45). Consequently, this specific type of alliance is likely to mitigate the bubble phenomenon.

Therefore, we propose that the overall positive effect posited in H1 is driven primarily by the bubble-inflating tendencies of horizontal and diversification alliances, while vertical alliances run counter to this trend. This leads to the following sub-hypotheses:

*Hypothesis 2a:* Horizontal strategic alliances are positively correlated with green innovation bubbles.

*Hypothesis 2b:* Vertical strategic alliances are negatively correlated with green innovation bubbles.

*Hypothesis 2c:* Diversification strategic alliances are positively correlated with green innovation bubbles.

### 2.3 The moderating role of new productive forces

The relationship between alliances and green innovation bubbles is unlikely to be uniform (46). We propose that a firm’s internal capabilities, captured by “new productive forces,” act as a critical moderator. New productive forces represent productivity driven by technological innovation and its deep integration with the real economy (22). Firms with high new productive forces possess strong absorptive capacity, a critical factor empirically linked to the success of R&D alliances (47, 48). For these firms, alliances are platforms for synergy, where external resources are effectively absorbed and converted into tangible outcomes (49). Their strong internal R&D foundation makes them less susceptible to free-riding and better able to leverage a partner’s contributions (19), thus mitigating bubble risk. Furthermore, such firms are more resilient to short-term market pressures (50) and more likely to pursue long-term innovation rather than symbolic actions (51). Conversely, firms with low new productive forces lack the ability to internalize alliance resources, making them more prone to the bubble phenomenon (52).

*Hypothesis 3:* New productive forces negatively moderate the relationship between corporate strategic alliances and green innovation bubbles.

### 2.4 Mediating mechanism analysis

#### 2.4.1 The mediating role of information asymmetry

Strategic alliances increase organizational opacity (20), making it harder for observers to assess a firm’s true green progress (53). This exacerbates information asymmetry. This is particularly relevant in the sustainability domain, where recent studies have empirically linked corporate opacity to a higher propensity for “greenwashing,” or symbolic environmentalism (54). Firms can leverage the alliance as an “information smokescreen,” signaling positive news while obscuring setbacks (55). As evidence shows that alliance announcements increase market volatility (56), investors may overvalue firms based on the alliance signal, fueling the bubble (57).

*Hypothesis 4a:* Information asymmetry plays a positive mediating role in the relationship between corporate strategic alliances and green innovation bubbles.

#### 2.4.2 The mediating role of policy resource acquisition

Alliances can be a powerful tool for securing government support (58) and building political connections to access subsidies for “dual carbon” goals (59). However, acquiring these resources can shift a firm’s focus. The objective can pivot from technological breakthroughs to satisfying bureaucratic requirements, a phenomenon known as “subsidy-driven innovation” (60). Cross-national studies showing that R&D subsidies do not uniformly translate into high-quality innovation outputs, particularly when monitoring is weak (61). This strategic reorientation toward optimizing grant proposals leads to a mismatch between policy resources received (input) and actual technological advancements (output), mediating the effect of alliances on the green innovation bubble (62).

*Hypothesis 4b:* Policy resource acquisition plays a positive mediating role in the relationship between corporate strategic alliances and green innovation bubbles.

**Figure 1:**
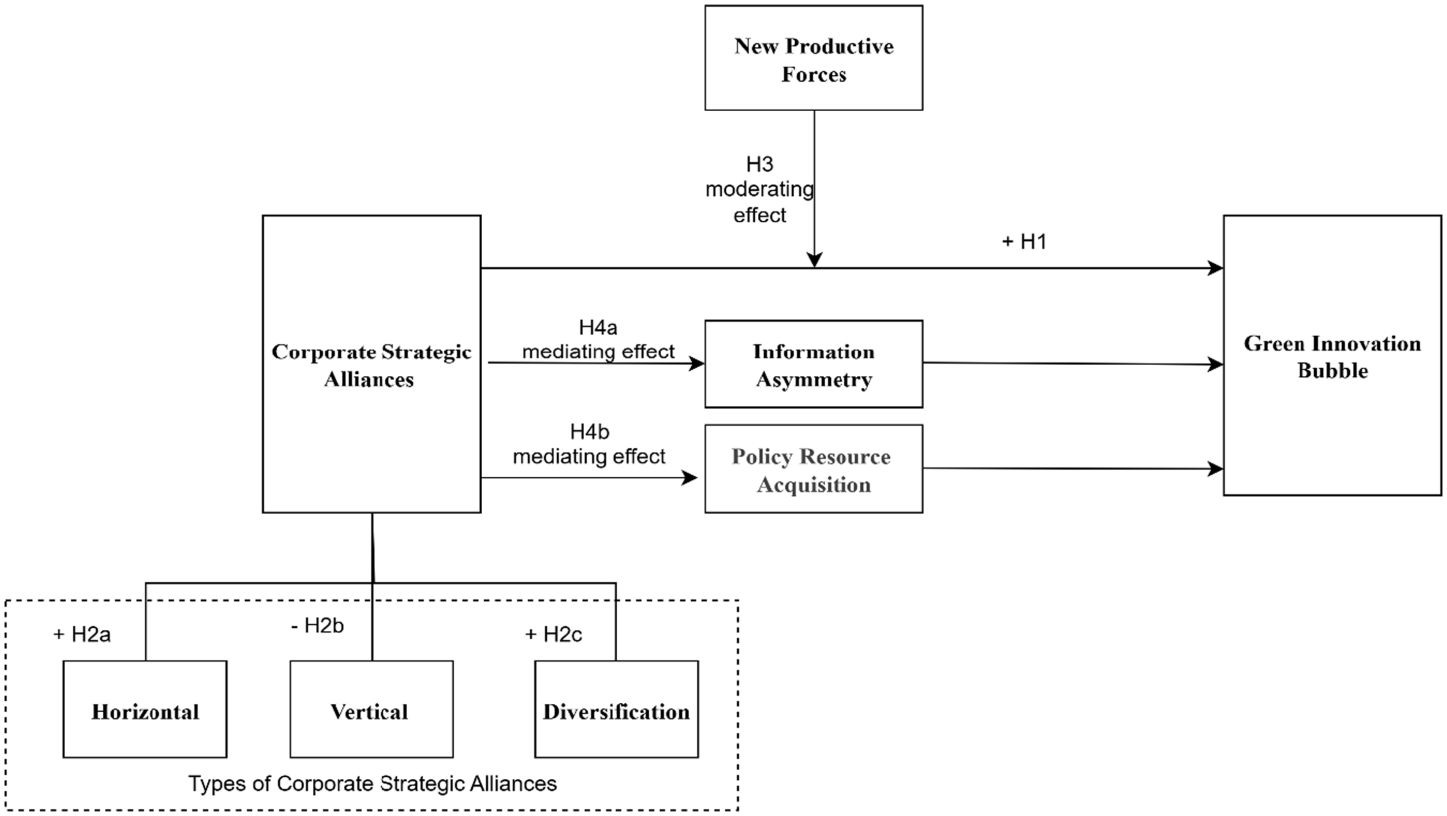
Theoretical framework diagram.

The theoretical framework diagram of this study is shown in Figure 1.

## 3 Research design

### 3.1 Sample selection and data sources

This study focuses on China’s A-share listed companies from 2015 to 2022. The following companies were excluded: (1) financial and insurance companies; (2) ST and *ST companies; (3) companies with significant data missing. The final balanced panel dataset includes 2,346 companies and 14,076 observations. This study focuses on Chinese A-share listed companies from 2015 to 2022. The following companies were excluded: (1) financial and insurance firms; (2) ST and *ST companies; and (3) companies with significant missing data. The final balanced panel dataset consists of 2,346 companies and 14,076 observations.

The data are obtained from multiple authoritative sources. Firm-level financial and governance information is primarily drawn from the CSMAR database, including:

Corporate Governance module: board size (Board) and ownership concentration (Top1, measured by the shareholding ratio of the largest shareholder).

Financial Indicators module: company size (Size, log of total assets), leverage (Lev, total liabilities/total assets), profitability (ROA, net profit/total assets), and growth (Growth, year-on-year operating revenue growth rate).

Innovation and R&D module: R&D investment (measured as R&D expenses disclosed in annual reports), number of R&D employees, and patent application data used for constructing new productive forces.

Mergers and Acquisitions/Alliances module: identification of strategic alliances. A dummy variable is coded as 1 if a company disclosed alliance participation in a given year, and 0 otherwise. Alliances are further classified into horizontal, vertical, and diversification types according to industry matching criteria.

Patent data are obtained from the National Intellectual Property Administration patent search system, with green patents identified using the WIPO IPC Green Inventory classification. Indicators such as green patent growth rate, average citation frequency, and proportion of invention patents are calculated to construct the Green Patent Bubble Index.

The WIND database is used to supplement financial statement information and to collect regional-level environmental investment data, which is employed to construct the environmental regulation (ER) variable.

Government subsidy data are drawn from the CSMAR Financial Statements and Subsidies module and cross-verified with corporate annual reports and social responsibility reports. Policy resource acquisition is measured by the ratio of government subsidies to non-operating income.

### 3.2 Variable definition and measurement

#### 3.2.1 Dependent variable: green patent bubble index (GPBI)

Following prior studies (63–65), we construct the Green Patent Bubble Index (GPBI Lite) to capture the potential mismatch between patenting activity and substantive technological contribution. A “bubble” in green innovation arises when patent activity expands rapidly in scale but lacks sufficient technological depth and structural support. To capture this imbalance, three complementary dimensions are employed:

Patent quantity expansion (GPG): an abnormal increase in the number of green patents may reflect speculative or policy-driven behavior, which is a potential signal of “excessive growth” beyond genuine innovation needs.

Technology quality shortage (GPI): measured by the average citation frequency of green invention patents. A lower citation frequency suggests weaker technological recognition and influence, highlighting a gap between innovation activity and substantive contribution.

Structural virtualization (GIF): the proportion of invention patents within total green patents. A lower share indicates that innovation relies more on utility models or designs, which generally embody lower technological content, suggesting a superficial structure of innovation.

In the comprehensive construction of the foam index, the three original indicators were first standardized (normalized to the range of 0–1) to eliminate dimensional differences. Later, the direction of foam was unified. The higher the patent growth rate, the greater the risk of foam. The lower the citation frequency and the proportion of inventions, the weaker the patent quality and technology content, and the higher the risk of foam. Therefore, GPG maintains its original direction, while GPI and GIF undergo reverse processing (1-normalization value). The value range of the index is [0,1], and the higher the value, the more obvious the “foam” feature of the enterprise in green patents, that is, the patent growth is too fast but the quality and structural support are insufficient, and there is a risk of false innovation or speculation.

#### 3.2.2 Independent variable: strategic alliance (SA)

The strategic alliance is measured using a dummy variable. If a company participates in a strategic alliance in a given year, the value is 1; otherwise, it is 0 (66, 67). Furthermore, the alliances are classified into three types: horizontal alliance (HSA), vertical alliance (VSA), and diversification alliance (DSA) (39).

#### 3.2.3 Mediating variables

(1) Information Asymmetry (InfoAsym): Following Barron et al. (68) and Huang et al. (69), we construct an information asymmetry index using principal component analysis (PCA). Specifically, we select three widely used proxies: (i) analyst forecast dispersion (the standard deviation of analysts’ EPS forecasts divided by the mean forecast), (ii) stock return volatility (standard deviation of daily returns), and (iii) turnover ratio (average daily trading volume/total shares outstanding). The first principal component extracted from these indicators is used as the composite index of information asymmetry. A higher value indicates greater information asymmetry.
(2) Policy Resource Acquisition (PolicyRes): Following Xu and Ruan (70), policy resource acquisition is measured by the ratio of government subsidies to a firm’s non-operating income. This indicator reflects the extent to which firms rely on government support for resources beyond their core business operations. A higher ratio suggests stronger dependence on policy resources. Consistent with Duan et al. (71), who demonstrate that different forms of subsidies (e.g., carbon reduction subsidies, remanufacturing subsidies, and consumer recycling subsidies) significantly influence firms’ strategic decisions in low-carbon innovation and supply chain management, we adopt subsidy-based measures as a reliable proxy for policy resource acquisition.

#### 3.2.4 Moderating variable: new productive forces (NQP)

New Productive Forces (NQP): Firms’ innovation capacity and sustainable competitiveness depend on the joint improvement of workforce quality and technological resources, which together drive productivity growth and green transformation. In addition, Chen et al. (29) highlight the role of government subsidies and policy resource acquisition in fostering corporate innovation, providing support for incorporating multi-dimensional inputs into the measurement of new productive forces. Following Song et al. (72), the new productive forces index is divided into two dimensions—labor and production tools—and the entropy method is employed to calculate the weight of each indicator. This reflects the idea that both human capital input and advanced technological tools are essential components of emerging productive forces. The specific indicators and their weighting results are reported in Table 1.

**Table 1:** Construction table of new quality productivity indicators.

First level indicator	Secondary indicators	Building content
Labor force	Proportion of R&D personnel salary	(R&D expenses—salary)/operating income
	Proportion of individuals with a bachelor’s degree	Number of undergraduate and above students/number of employees
	Proportion of R&D personnel	Number of R&D personnel/number of employees
Subject of labor	Proportion of fixed assets	Fixed assets/total assets
	Proportion of manufacturing costs	(Subtotal of cash outflows from operating activities + depreciation of fixed assets + amortization of intangible assets + provision for impairment—cash paid for goods purchased and services received—cash paid to and for employees)/(Subtotal of cash outflows from operating activities + depreciation of fixed assets + amortization of intangible assets + provision for impairment)
	Proportion of environmental investment	Environmental investment/total assets
Means of labor	Proportion of R&D depreciation and amortization	(R&D expenses—depreciation and amortization)/operating income
	Proportion of R&D leasing	(R&D expenses—rental fees)/operating income
	Proportion of direct R&D investment	(R&D expenses—direct investment)/operating income
	Total Asset turnover	Operating revenue/average total assets
	Reciprocal of equity multiplier	Owner’s equity/total assets

#### 3.2.5 Control variables

Based on relevant literature, we include a set of firm-level, governance, and external environment characteristics to mitigate potential confounding effects. These variables have been widely recognized as determinants of firm performance and innovation outcomes (73, 74). In addition, following Hünermund and Louw (75), we acknowledge the importance of carefully accounting for potential nuisance effects when including controls in causal regression analysis. Year and industry fixed effects are also included to capture unobserved time-varying and sector-specific heterogeneity:

(1) Company Size (Size): The natural logarithm of total assets;
(2) Company Age (Age): The natural logarithm of the number of years since the company’s establishment;
(3) Debt-to-Asset Ratio (Lev): Total liabilities divided by total assets;
(4) Profitability (ROA): Net profit divided by total assets;
(5) Growth (Growth): Operating revenue growth rate;
(6) Ownership Concentration (Top1): The proportion of shares held by the largest shareholder;
(7) Government Subsidy (Subsidy): Government subsidies divided by operating income;
(8) Institutional Investor Ownership (Inst): The total proportion of shares held by institutional investors;
(9) Board Size (Board): The number of members on the board of directors;
(10) Industry Competition Intensity (HHI): The Herfindahl–Hirschman Index;
(11) Environmental Regulation Intensity (ER): Environmental investment in the company’s region divided by GDP.

Year and industry fixed effects are also Controlled for.

### 3.3 Model specification

To empirically test the research hypotheses, we construct the following panel regression models. All models include firm-level control variables and year and industry fixed effects to account for unobserved heterogeneity and time-/sector-specific shocks. Robust standard errors clustered at the firm level are used to address potential heteroskedasticity and autocorrelation:

Model 1 tests the direct impact of strategic alliances (SA) on green innovation bubbles (GPBI):


$${\text{GPBI}}_{i,t}=\beta_{0}+\beta_{1}SA_{i,t}+\Sigma \beta_{k}\text{Contro}l_{i,t}+\varepsilon_{i,t}$$

Model 2 incorporates the moderating effect of new productive forces (NQP) on the SA–GPBI relationship:


$$\begin{array}{l} {\text{GPBI}}_{i,t}=\beta_{0}+\beta_{1}SA_{i,t}+\beta_{2}\mathrm{NQ}P_{i,t}+\beta_{3}SA_{i,t}\times \mathrm{NQ}P_{i,t}+\\ \Sigma \beta_{k}\text{Contro}l_{i,t}+\varepsilon_{i,t} \end{array}$$

Model 3 decomposes strategic alliances into three subdimensions—horizontal (HSA), vertical (VSA), and diversified (DSA)—to test their heterogeneous effects on green innovation bubbles:


$$\begin{array}{l} {\text{GPBI}}_{i,t}=\beta_{0}+\beta_{1}\mathrm{HS}A_{i,t}+\beta_{2}\mathrm{VS}A_{i,t}+\beta_{3}\mathrm{DS}A_{i,t}+\\ \Sigma \beta_{k}\text{Contro}l_{i,t}+\varepsilon_{i,t} \end{array}$$

Where *i* represents the company, t represents the year, Control represents the set of Control variables, and *ε* is the random error term.

## 4 Empirical results analysis

### 4.1 Descriptive statistics

Table 2 presents the descriptive statistics of the main variables. The mean value of the Green Patent Bubble Index (GPBI) is 1.247, with a standard deviation of 0.863, indicating considerable variation in the level of green innovation bubbles among the sample companies. The mean value of strategic alliances (SA) is 0.326, suggesting that approximately 32.6% of the sample companies participated in strategic alliances. The mean value of new productive forces (NQP) is 0.483, with a standard deviation of 0.215, indicating significant variation in the level of new productive forces among the sample companies. The mean value of information asymmetry (InfoAsym) is 0.386, and the mean value of policy resource acquisition (PolicyRes) is 0.012, both showing considerable sample variation.

**Table 2:** Descriptive statistics.

Variable	Observations	Average value	Standard deviation	Minimum value	Median	Maximum value
GPBI	14,076	1.247	0.863	0.105	1.132	4.876
SA	14,076	0.326	0.469	0	0	1
HSA	14,076	0.156	0.363	0	0	1
VSA	14,076	0.098	0.297	0	0	1
DSA	14,076	0.072	0.259	0	0	1
NQP	14,076	0.483	0.215	0.068	0.462	0.927
InfoAsym	14,076	0.386	0.173	0.087	0.365	0.892
PolicyRes	14,076	0.012	0.018	0	0.007	0.124
Size	14,076	22.563	1.342	19.876	22.418	26.943
Age	14,076	2.873	0.417	1.099	2.944	3.761
Lev	14,076	0.437	0.196	0.054	0.426	0.891
ROA	14,076	0.048	0.053	−0.176	0.042	0.213
Growth	14,076	0.186	0.437	−0.562	0.124	2.743
Top1	14,076	34.672	14.539	8.763	32.845	75.264
Subsidy	14,076	0.012	0.018	0	0.007	0.124
Inst	14,076	46.853	23.476	3.254	48.672	89.435
Board	14,076	8.736	1.842	5	9	15
HHI	14,076	0.087	0.092	0.012	0.063	0.487
ER	14,076	0.023	0.011	0.005	0.022	0.058

### 4.2 Correlation analysis

Table 3 presents the Pearson correlation coefficients between the main variables. SA are significantly positively correlated with the GPBI (*r* = 0.163, *p* < 0.01), which provides preliminary support for Hypothesis 1. NQP are significantly negatively correlated with the green innovation bubble (GPBI) (*r* = −0.215, *p* < 0.01). SA is significantly positively correlated with information asymmetry (InfoAsym) (*r* = 0.146, *p* < 0.01) and PolicyRes (*r* = 0.173, *p* < 0.01). Both InfoAsym and PolicyRes are significantly positively correlated with GPBI, with correlation coefficients of 0.187 (*p* < 0.01) and 0.195 (*p* < 0.01), respectively, providing preliminary support for Hypotheses 4a and 4b. The absolute values of the correlation coefficients between all variables are less than 0.7, indicating that multicollinearity is not a serious issue.

**Table 3:** Correlation analysis.

Variable	GPBI	SA	NQP	InfoAsym	PolicyRes	Size	Age	Lev	ROA
GPBI	1								
SA	0.163***	1							
NQP	−0.215***	0.142***	1						
InfoAsym	0.187***	0.146***	−0.167***	1					
PolicyRes	0.195***	0.173***	−0.084***	0.092***	1				
Size	−0.087***	0.196***	0.224***	−0.153***	0.047**	1			
Age	0.046**	0.073***	−0.052**	0.038*	0.065***	0.247***	1		
Lev	0.103***	0.087***	−0.063***	0.076***	0.084***	0.435***	0.176***	1	
ROA	−0.156***	0.045**	0.283***	−0.142***	−0.037*	−0.087***	−0.126***	−0.342***	1
Growth	0.032	0.063***	0.126***	0.089***	0.053**	0.073***	−0.132***	0.028	0.247***
Top1	−0.058**	0.024	0.087***	−0.076***	−0.042**	0.163***	−0.053**	−0.065***	0.126***
Subsidy	0.187***	0.168***	−0.073***	0.083***	0.683***	0.043**	0.062***	0.078***	−0.026
Inst	−0.073***	0.053**	0.186***	−0.167***	−0.058**	0.237***	0.048**	−0.047**	0.173***
Board	−0.042**	0.036*	0.047**	−0.034*	0.023	0.216***	0.127***	0.082***	0.035*
HHI	0.063***	−0.027	−0.058**	0.046**	0.037*	−0.047**	0.026	0.032	−0.067***
ER	−0.076***	−0.032	0.147***	−0.053**	−0.068***	0.036*	−0.023	−0.037*	0.057**

Variable	Growth	Top1	Subsidy	Inst	Board	HHI	ER
Growth	1						
Top1	0.043**	1					
Subsidy	0.047**	−0.037*	1				
Inst	0.082***	0.126***	−0.052**	1			
Board	0.018	0.073***	0.017	0.057**	1		
HHI	−0.083***	−0.028	0.043**	−0.036*	0.018	1	
ER	0.042**	0.026	−0.073***	0.062***	0.024	−0.043**	1

*, **, *** denote significance at the 10, 5, and 1% levels, respectively.

### 4.3 Regression analysis results

Column (1) of Table 4 reports the regression results for Model 1. The coefficient of strategic alliances (SA) is 0.176, which is significantly positive at the 1% level, indicating that corporate participation in strategic alliances significantly increases the green innovation bubble, supporting Hypothesis 1. This result highlights the economic implications of strategic alliances: while these partnerships may signal green innovation efforts, they could also be a tool for firms to gain policy support or market recognition without substantial technological improvements, creating a mismatch between innovation input and output.

**Table 4:** Regression results of corporate strategic alliances and green innovation bubble.

Variable	(1) GPBI	(2) GPBI	(3) GPBI
SA	0.176***	0.168***	
	(4.27)	(4.15)	
NQP		−0.387***	−0.392***
		(−6.83)	(−6.92)
SA × NQP		−0.253***	
		(−3.46)	
HSA			0.213***
			(4.56)
VSA			−0.142**
			(−2.38)
DSA			0.167***
			(3.24)
Size	−0.052**	−0.043*	−0.046**
	(−2.18)	(−1.84)	(−1.97)
Age	0.087**	0.083**	0.085**
	(2.36)	(2.27)	(2.31)
Lev	0.263***	0.257***	0.259***
	(3.42)	(3.37)	(3.39)
ROA	−0.875***	−0.763***	−0.768***
	(−4.23)	(−3.74)	(−3.76)
Growth	0.042	0.036	0.037
	(1.26)	(1.09)	(1.12)
Top1	−0.003**	−0.003**	−0.003**
	(−2.47)	(−2.43)	(−2.45)
Subsidy	1.246**	1.187**	1.192**
	(2.18)	(2.09)	(2.10)
Inst	−0.002**	−0.002**	−0.002**
	(−2.35)	(−2.31)	(−2.33)
Board	−0.016*	−0.015*	−0.015*
	(−1.83)	(−1.76)	(−1.78)
HHI	0.437**	0.426**	0.428**
	(2.42)	(2.37)	(2.38)
ER	−1.873**	−1.826**	−1.835**
	(−2.15)	(−2.11)	(−2.12)
Constant term	2.463***	2.647***	2.658***
	(4.87)	(5.26)	(5.28)
Industry fixed effects	Control	Control	Control
Observations	14,076	14,076	14,076
*R* ^2^	0.186	0.203	0.207
Adjust *R*^2^	0.179	0.195	0.199
*F*	16.83***	17.46***	17.62***

The values in parentheses are *t*-values; *, **, *** denote significance at the 10, 5, and 1% levels, respectively.

Column (2) of Table 4 shows that the coefficient of the interaction term SA × NQP is −0.253, which is significantly negative at the 1% level, indicating that new productive forces negatively moderate the relationship between corporate strategic alliances and the green innovation bubble, supporting Hypothesis 3. This suggests that companies with high levels of new productive forces are able to effectively utilize strategic alliance resources and reduce the risk of a green innovation bubble.

Column (3) of Table 4 shows that the coefficient of horizontal alliances (HSA) is 0.213 and the coefficient of diversification alliances (DSA) is 0.167, both significantly positive at the 1% level, supporting Hypotheses 2a and 2c. The coefficient of vertical alliances (VSA) is −0.142, significantly negative at the 5% level, supporting Hypothesis 2b. This indicates that different types of strategic alliances have significantly different impacts on the green innovation bubble.

### 4.4 Mediation effect test

Table 5 reports the results of the mediation effect test for information asymmetry and policy resource acquisition. The bootstrap method was used to conduct the mediation effect test. The total effect of corporate strategic alliances on the green innovation bubble is 0.176 (*p* < 0.01), with a direct effect of 0.078 (*p* < 0.05), accounting for 44.3% of the total effect; the total indirect effect is 0.098 (*p* < 0.01), accounting for 55.7% of the total effect. Specifically, the mediation effect of information asymmetry is 0.040 (*p* < 0.01), accounting for 22.7% of the total effect; the mediation effect of policy resource acquisition is 0.034 (*p* < 0.01), accounting for 19.3% of the total effect. Additionally, there is a chain mediation path “SA → InfoAsym → PolicyRes → GPBI” with an effect value of 0.024 (*p* < 0.01), accounting for 13.7% of the total effect. The bootstrap 95% confidence intervals for all mediation effects do not include 0, indicating that the mediation effects are significant. These results support Hypotheses 4a and 4b, which state that information asymmetry and policy resource acquisition play a significant positive mediating role in the relationship between strategic alliances and green innovation bubbles.

**Table 5:** Mediation effect test.

Route	Effect value	Standard error	Bootstrap 95% CI	Proportion of total effect	Return steps	Regression coefficient	*T*	Sobel *Z*	Sobel *p*
Total effect SA → GPBI	0.176***	0.041	[0.096, 0.256]	100%	Step 1	0.176***	4.27	—	—
direct effect SA → GPBI	0.078**	0.031	[0.017, 0.139]	44.3%	Step 3	0.078**	2.52	—	—
Indirect effect 1 (SA → InfoAsym → GPBI)	0.040***	0.012	[0.018, 0.064]	22.7%	Step 2a Step 3a	0.146*** 0.301***	3.80 5.52	3.68	<0.001
Indirect effect 2 (SA → PolicyRes → GPBI)	0.034***	0.009	[0.017, 0.053]	19.3%	Step 2b Step 3b	0.173*** 0.212***	4.01 5.96	3.41	<0.001
Indirect effect 3 (SA → InfoAsym → PolicyRes → GPBI)	0.024***	0.007	[0.011, 0.039]	13.7%	Step 2a Step 2c Step 3b	0.146*** 0.128*** 0.212***	3.80 3.22 5.96	3.02	0.002
Total indirect effects	0.098***	0.023	[0.054, 0.144]	55.7%	—	—	—	—	—

*, **, *** denote significance at the 10, 5, and 1% levels, respectively. The three-step regression methods are Step 1 (SA → GPBI), Step 2 (SA → mediator variable), and Step 3 (SA + mediator → GPBI); Sobel *Z*-value is estimated based on the two-stage regression coefficients and standard errors of the indirect path.

### 4.5 Robustness test

To mitigate potential endogeneity issues, an instrumental variable method was employed for testing. Geographic proximity of corporate strategic alliances (76) was selected as the instrumental variable, and a two-stage least squares (2SLS) regression was conducted. Column (1) of Table 6 shows that the Hausman test results support the use of the instrumental variable, and the first-stage *F*-statistic is greater than 10, indicating that the instrumental variable does not suffer from the weak instrument problem. The second-stage regression results are consistent with the main regression results, suggesting that the research conclusions are robust.

**Table 6:** Robustness test.

Variable	(1) 2SLS	(2) Substitution variable			(3) Eliminate extreme values	(4) Fixed panel effect
		Citation Ratio	Alliance Freq	TFP		
SA	0.193***	0.158***	0.169***	0.134**	0.169***	0.147***
	(3.86)	(3.97)	(4.18)	(2.12)	(4.18)	(3.62)
NQP	−0.402***	−0.376***	−0.383***	−0.358***	−0.383***	−0.358***
	(−6.95)	(−6.74)	(−6.79)	(−6.42)	(−6.79)	(−6.42)
SA × NQP	−0.267***	−0.241***	−0.383***	−0.232***	−0.248***	−0.232***
	(−3.53)	(−3.38)	(−6.79)	(−3.17)	(−3.42)	(−3.17)
InfoAsym	0.438***	0.421***	0.389***	0.417***	0.432***	0.417***
	(5.52)	(5.37)	(5.12)	(5.26)	(5.43)	(5.26)
PolicyRes	5.257***	5.183***	5.216***	5.124***	5.216***	5.124***
	(5.96)	(5.87)	(5.92)	(5.78)	(5.92)	(5.78)
Control variable	Control	Control	Control	Control	Control	Control
Fixed year effect	Control	Control	Control	Control	Control	Control
Industry fixed effects	Control	Control	Control	Control	Control	–
Fixed effects of enterprises	–	–	–	–	–	Control
Observations	14,076	14,076	14,076	14,076	13,794	14,076
*R* ^2^	0.248	0.242	0.251	0.239	0.251	0.236
Hausman *p*	0.023					
Phase 1 *F*-statistic	24.76					
Sargan’s over identification test *p*-value	0.259					

The values in parentheses are *t*-values. *, **, *** denote significance at the 10, 5, and 1% levels, respectively.

To further validate the robustness of our findings and the rationality of the variable construction, we conducted a series of sensitivity analyses using alternative indicators.

First, for the dependent variable, the Green Patent Bubble Index (GPBI Lite) was replaced with the ratio of firm-level green patent citation rates relative to the industry average. This alternative indicator, following Yang et al. (77) and Fang and Li (78), captures the quality dimension of green innovation more directly and has been widely used in distinguishing high-quality innovation from innovation bubbles.

Second, for the independent variable (strategic alliances, SA), instead of a binary indicator, we used the number of strategic cooperation agreements disclosed by each firm in a given year. This approach is consistent with Goerzen (79), who argues that repeated and multiple partnerships better reflect the intensity and depth of alliance networks.

Third, for the moderating variable (new productive forces, NQP), we substituted the baseline index with total factor productivity (TFP). This follows Gao and Li (80), who link new quality productive forces with carbon-related productivity measures, and provides an alternative perspective on firm-level technological capacity.

The results of these alternative regressions, reported in Column (2) of Table 6, remain consistent with the baseline estimates in both sign and significance. This robustness check indicates that our conclusions are not sensitive to the specific measurement choices of GPBI Lite or the other core variables, thereby enhancing the credibility of the analysis.

After removing extreme value samples (the top and bottom 1%), a re-estimation was performed, with results shown in Column (3) of Table 6. The main conclusions remain unchanged. Furthermore, a panel fixed-effect model was used to Control for unobservable firm heterogeneity, with results shown in Column (4) of Table 6. The main conclusions remain robust.

### 4.6 Further analysis

The sample was divided into state-owned and non-state-owned enterprises based on ownership type, and into high environmental regulation and low environmental regulation groups based on the median of environmental regulation intensity. Regressions were conducted for each group, with results shown in Table 7. The positive impact of strategic alliances on the green innovation bubble is more significant in state-owned enterprises (*β* = 0.215, *p* < 0.01), while the impact is weaker in non-state-owned enterprises (*β* = 0.143, *p* < 0.05), with a significant difference (*p* < 0.05). This may be because state-owned enterprises are more likely to access policy resources and have a stronger motivation to convey green signals through strategic alliances. The mediating effect of policy resource acquisition is also significantly stronger in state-owned enterprises than in non-state-owned enterprises (5.683 vs. 4.876, *p* < 0.05), further supporting this explanation. In regions with high environmental regulation intensity, the positive impact of strategic alliances on the green innovation bubble is weaker (*β* = 0.132, *p* < 0.05); in regions with low environmental regulation intensity, this effect is more significant (*β* = 0.207, *p* < 0.01), with a significant difference (*p* < 0.05). This indicates that strict environmental regulation can suppress the green innovation bubble. The mediating effect of policy resource acquisition is also significantly stronger in low environmental regulation areas than in high environmental regulation areas (5.642 vs. 4.876, *p* < 0.05), suggesting that environmental regulation can reduce the tendency for companies to form green innovation bubbles through policy resource acquisition.

**Table 7:** Further analysis results.

Variable	(1) State-owned enterprise	(2) Non state-owned enterprises	(1) High environmental regulations	(2) Low environmental regulations
SA	0.215***	0.143**	0.132**	0.207***
	(4.53)	(2.37)	(2.41)	(4.62)
NQP	−0.425***	−0.356***	−0.412***	−0.363***
	(−6.92)	(−5.84)	(−6.87)	(−5.92)
SA × NQP	−0.287***	−0.218***	−0.285***	−0.227***
	(−3.76)	(−2.98)	(−3.73)	(−3.12)
InfoAsym	0.458***	0.417***	0.426***	0.453***
	(5.62)	(5.27)	(5.32)	(5.57)
PolicyRes	5.683***	4.876***	4.876***	5.642***
	(6.13)	(5.42)	(5.42)	(6.08)
Control variable	Control	Control	Control	Control
Fixed year effect	Control	Control	Control	Control
Industry fixed effects	Control	Control	Control	Control
Observations	6,842	7,234	7,038	7,038
*R* ^2^	0.213	0.176	0.195	0.209

The values in parentheses are *t*-values. *, **, *** denote significance at the 10, 5, and 1% levels, respectively.

To explore the interaction between different types of strategic alliances and new productive forces, we constructed interaction terms and performed a regression analysis.

Table 8 shows that new productive forces significantly reduce the green innovation bubble effects in horizontal and diversification alliances (HSA × NQP = −0.287, *p* < 0.01; DSA × NQP = −0.243, *p* < 0.01). This indicates that firms with stronger innovative capabilities can mitigate the risks of superficial innovation in these alliances, leading to more substantial green technologies. However, we also observe a slight positive moderating effect on vertical alliances (VSA × NQP = 0.124, *p* < 0.1), suggesting that while vertical alliances typically drive meaningful innovation, the presence of new productive forces may slightly diminish their effectiveness in this context. This highlights the need for a balanced approach, where strong innovation capabilities are crucial for maximizing the impact of different types of strategic alliances in addressing environmental and public health challenges.

**Table 8:** Interaction between different types of strategic alliances and new productive forces.

Variable	(1) GPBI
HSA	0.225***
	(4.67)
VSA	−0.153**
	(−2.46)
DSA	0.176***
	(3.35)
NQP	−0.396***
	(−6.95)
HSA × NQP	−0.287***
	(−3.76)
VSA × NQP	0.124*
	(1.85)
DSA × NQP	−0.243***
	(−3.27)
InfoAsym	0.435***
	(5.47)
PolicyRes	5.273***
	(5.98)
Control variable	Control
Fixed year effect	Control
Industry fixed effects	Control
Observations	14,076
*R* ^2^	0.218

The values in parentheses are *t*-values. *, **, *** denote significance at the 10, 5, and 1% levels, respectively.

## 5 Conclusions and implications

### 5.1 Conclusion

In this study, we explore the relationship between corporate strategic alliances, green innovation bubbles, and new productive forces, using data from China’s A-share listed companies between 2015 and 2022. Our findings provide valuable insights into the complexities of green innovation and its implications for both businesses and public health.

We find that while strategic alliances can facilitate collaboration and resource sharing, they can also unintentionally contribute to the creation of green innovation bubbles. This happens when firms form alliances primarily to signal their green commitment rather than to drive meaningful technological breakthroughs. As a result, resources are often misallocated, delaying the deployment of effective green technologies and hindering progress on critical environmental health outcomes, such as reducing air pollution and mitigating climate-related health risks.

Our research also shows that companies with stronger new productive forces—advanced technological capabilities that are integrated with the real economy—are in a better position to transform alliance resources into real, impactful green innovations. These firms are less likely to fall into the trap of superficial innovations. On the other hand, companies with fewer productive capabilities may struggle to turn alliances into tangible results, which further perpetuates the green innovation bubble and delays the societal benefits that could come from genuine technological advancements.

We also highlight the role of information asymmetry and policy resource acquisition in shaping green innovation bubbles. When firms focus more on securing policy benefits—like subsidies—rather than fostering true innovation, it can lead to a misdirection of resources, slowing down the progress toward substantial technological breakthroughs that could improve public health outcomes.

Furthermore, our study reveals that different types of strategic alliances—horizontal, vertical, and diversification—have varying effects on green innovation. Horizontal and diversification alliances tend to inflate green innovation bubbles, while vertical alliances, which are more aligned with supply chains, often lead to more meaningful innovation. This reinforces the importance of carefully designed strategic alliances that prioritize tangible, market-driven solutions to environmental problems.

In conclusion, our research emphasizes the need for both businesses and policymakers to focus on promoting genuine green innovations. For businesses, it’s crucial to form alliances that drive real, measurable environmental change, rather than merely signaling sustainability. For policymakers, targeted regulations and resource allocation that support substantial innovation are key to ensuring that green technologies deliver long-term public health benefits. Ultimately, fostering authentic green innovations will not only improve environmental outcomes but also create lasting societal value.

### 5.2 Theoretical contributions

This study makes several theoretical contributions to the existing literature:

First, it complements and extends existing research on the innovation outcomes of strategic alliances. While prior studies have largely emphasized the positive impact of alliances on innovation performance (10, 81), recent discussions have called for a more nuanced understanding of alliance risks (82). By showing that strategic alliances may lead to the formation of green innovation bubbles—characterized by a mismatch between innovation quantity and quality—this study highlights a potential downside of alliances in specific institutional contexts. This finding helps refine the boundary conditions under which alliances contribute to innovation and adds empirical evidence to the emerging discourse on the “dark side” of inter-organizational collaboration.

Second, our study introduces the concept of new productive forces into the strategic management literature and empirically examines its moderating role. Although this concept has appeared in policy and macroeconomic discourse, its implications for firm-level innovation behavior remain underexplored. By identifying new productive forces as a contingent factor that shapes how alliances influence innovation quality, this study responds to calls to integrate broader developmental constructs into firm strategy research (83), offering a novel lens for explaining heterogeneity in alliance outcomes.

Third, our research contributes to the understanding of green innovation inefficiency by clarifying the micro-level mechanisms underlying green innovation bubbles. While previous literature has recognized the phenomenon (84), its internal drivers remain insufficiently theorized. By identifying information asymmetry and policy resource acquisition as mediating variables, this study adds explanatory depth and integrates insights from resource dependence theory and signaling theory, thereby enriching the theoretical foundations of green innovation research (85).

Fourth, our study provides a more refined view of strategic alliances by differentiating between horizontal, vertical, and diversification alliances. Existing literature often treats alliances as a homogeneous construct, overlooking internal structural variation (86). By comparing how different alliance types affect the formation of green innovation bubbles, this research underscores the importance of alliance configuration in shaping innovation outcomes and contributes to the growing on alliance typologies.

### 5.3 Practical implications

For corporate managers, our results suggest that the strategic intent behind forming a green alliance must be matched with an appropriate structure. The finding that vertical alliances are negatively correlated with innovation bubbles indicates that managers should prioritize close collaboration with supply chain partners to solve tangible environmental problems. This structure grounds R&D in practical applications and market demands, such as developing circular material flows or reducing Scope 3 emissions, which is more likely to lead to measurable improvements in environmental and public health outcomes. In contrast, managers should be cautious when entering horizontal or diversification alliances due to their positive association with innovation bubbles. For these partnerships, there is a significant risk that competitive pressures or the desire to signal innovation leadership can lead to a focus on public perception rather than on tangible outcomes, thereby failing to address real environmental health risks. To mitigate this, firms should establish clear governance mechanisms, set specific technical milestones, and enhance transparency by reporting on environmental performance metrics, not just R&D expenditures. This helps align the alliance’s activities with genuine innovation and builds credibility with stakeholders.

For policymakers, this study suggests that broad, non-differentiated support for all types of green alliances may be an inefficient use of public funds. Our finding that different alliance types produce divergent outcomes calls for a more targeted policy approach. First, government incentives, such as subsidies or tax credits, could be designed to preferentially support vertical, supply-chain-level collaborations, as these are shown to be more effective in preventing innovation bubbles. This encourages partnerships that are focused on practical, systemic solutions. Second, when providing support for higher-risk horizontal and diversification alliances, policy should shift from input-based subsidies (e.g., funding R&D spending) to outcome-based incentives. For example, funding could be disbursed in tranches, conditional on the alliance achieving specific, pre-agreed environmental performance targets. This ensures that public resources are tied to verifiable progress. Finally, given our finding that bubbles are more pronounced in regions with weaker environmental regulation, a uniform national policy may be inadequate. Policymakers should consider implementing stricter monitoring and third-party auditing requirements for alliance projects in such regions to ensure that public investments generate real environmental improvements and their associated public health co-benefits.

For investors, the signaling dynamics uncovered in our study indicate that alliance announcements, particularly in policy-sensitive sectors, may not always reflect substantive technological advancement. Investors should incorporate quality-adjusted innovation indicators—such as patent citation rates or invention patent shares—into their due diligence processes and pay closer attention to firms’ underlying innovation capacity. Firms with higher levels of new productive forces are more likely to translate external partnerships into durable environmental and social value. By focusing on innovation that yields demonstrable outcomes—such as lower emissions intensity or improved environmental compliance—investors can better align financial objectives with broader sustainability goals, including those relevant to public health protection.

### 5.4 Research limitations and future directions

Although this study provides valuable findings, it still has some limitations, which also provide directions for future research:

First, in terms of measurement, our proxy for green innovation bubbles—though grounded in prior literature—relies primarily on patent-related indicators, such as growth rate, structural composition, and citation frequency. While these measures capture aspects of innovation inflation, they may not fully reflect the real-world environmental or health impact of green technologies. Future research could develop multi-dimensional evaluation systems that integrate patent data with market-based outcomes (e.g., stock reactions, carbon performance, ESG scores), or include third-party sustainability certifications and pollution abatement records to construct more externally validated bubble indices.

Second, with regard to research design, our study uses panel regression models based on observational data, which, despite robustness checks (e.g., instrumental variables, fixed effects), cannot fully eliminate endogeneity concerns. To address this, future studies could apply quasi-natural experiments—such as policy shocks or staggered alliance reforms—to better infer causality. Additionally, event study methodologies could be used to assess market perceptions of alliance announcements, and dynamic panel models (e.g., system GMM) could help capture temporal feedback effects between strategic alliances and innovation output quality.

Third, in terms of contextual and sample limitations, this study focuses solely on Chinese A-share listed companies, which operate in a unique institutional environment with strong policy signals and active state intervention. This may limit the generalizability of findings to other countries or to unlisted firms. Future research could explore cross-country comparisons to examine how institutional quality, regulatory stringency, or environmental governance models moderate the relationship between strategic alliances and innovation bubbles. Expanding the sample to include SMEs or privately held firms could also shed light on alliance behavior and innovation quality under different resource constraints.

Fourth, from a theoretical perspective, although we identify key mechanisms such as information asymmetry and policy resource acquisition, other potential pathways remain underexplored. Future studies could investigate additional mediators such as corporate social responsibility orientation, board environmental expertise, or external ESG rating pressure. Similarly, moderation effects from digital transformation, organizational learning capabilities, or environmental risk exposure could offer richer insight into when and how green alliances turn symbolic. Moreover, while our study introduces “new productive forces” as a novel moderator, future research could delve deeper into its composition, measurement heterogeneity across industries, or its interplay with national innovation policies.

## Data Availability

The data analyzed in this study is subject to the following licenses/restrictions: Data Availability Statement: The datasets generated and analyzed during this study are derived from the following sources: CSMAR Database: Financial and governance data for Chinese A-share listed companies. WIND Database: Supplementary financial and market data. National Intellectual Property Administration Patent Retrieval System: Green patent application and authorization records. Corporate Annual Reports and Social Responsibility Reports: Manually collected and collated firm-level disclosures. Due to licensing restrictions, the raw data cannot be publicly shared but are available from the first author upon reasonable request for academic purposes. Requests to access these datasets should be directed to 18678660323@163.com.
